# SARS-CoV-2 infection and genomic diversity in Quilombola communities during Brazil’s gamma VOC wave

**DOI:** 10.1016/j.isci.2026.118113

**Published:** 2026-11-05

**Authors:** Filipe Ferreira de Almeida Rego, Luciane Amorim Santos, Marina Silveira Cucco, Laise de Moraes, Pilar Tavares Veras Florentino, Juracy Bertoldo Junior, Vinícius de Araújo Oliveira, Jorge Sadao Nihei, Fernando Vicentini, Jeiza Botelho Leal Reis, Catia Fernandes, Guangdi Li, Camila I. de Oliveira, Hermes Pedreira da Silva Filho, Ricardo Khouri

**Affiliations:** 1 Gonçalo Moniz Institute, Oswaldo Cruz Foundation (Fiocruz), Salvador, BA, Brazil; 2 Escola Bahiana de Medicina e Saúde Pública (EBMSP), Salvador, BA, Brazil; 3 DigiSaude - National Institute of Science and Technology in Digital Health, Salvador, BA, Brazil; 4 Centre of Data and Knowledge Integration for Health (CIDACS), Instituto Gonçalo Moniz Institute, Oswaldo Cruz Foundation (Fiocruz), Salvador, BA, Brazil; 5 Universidade Federal do Recôncavo da Bahia (UFRB), Santo Antônio de Jesus, BA, Brazil; 6 Secretaria Municipal de Saúde de Cachoeira, Cachoeira, BA, Brazil; 7 Hunan Provincial Key Laboratory of Clinical Epidemiology, Xiangya School of Public Health, Central South University, Changsha, China; 8 INCT-Doenças Tropicais, Salvador, BA, Brazil; 9 Faculdade de Medicina, Universidade Federal da Bahia (UFBA), Salvador, BA, Brazil

**Keywords:** SARS-CoV-2, COVID-19, Quilombola communities, genomic surveillance, health disparities

## Abstract

COVID-19 has disproportionately affected vulnerable populations worldwide; yet, data on SARS-CoV-2 transmission and variant diversity in Brazil’s descendants of enslaved individuals, known as Quilombola, remain scarce. We conducted a cross-sectional study in Cachoeira, Bahia, in people seeking care, between February and March 2021, testing 700 oropharyngeal swabs by RT-qPCR and 591 plasma samples for IgM/IgG antibodies. Positive samples with Cq < 25 underwent genomic sequencing and phylogenetic analysis. SARS-CoV-2 infection burden was comparable between urban (31.4%) and rural (32.0%) zones in Quilombola communities. Genomic analysis of 39 sequences identified multiple lineages, predominantly the Gamma variant (P.1), with phylogenetic evidence of numerous introductions and both local and external transmission. These results reveal complex transmission dynamics and variant diversity within marginalized Quilombola populations, underscoring the need for targeted public health interventions, expanded diagnostic coverage, and prioritization of these communities in vaccination campaigns to address health inequities.

## Highlights

• SARS-CoV-2 infection burden was similar in urban and rural Quilombola areas
• Gamma variant (P.1) predominated among sequenced SARS-CoV-2 samples
• Multiple lineages and introductions show complex SARS-CoV-2 transmission
• Targeted interventions and vaccination are needed for Quilombola equity

## Graphical abstract

**Figure:**
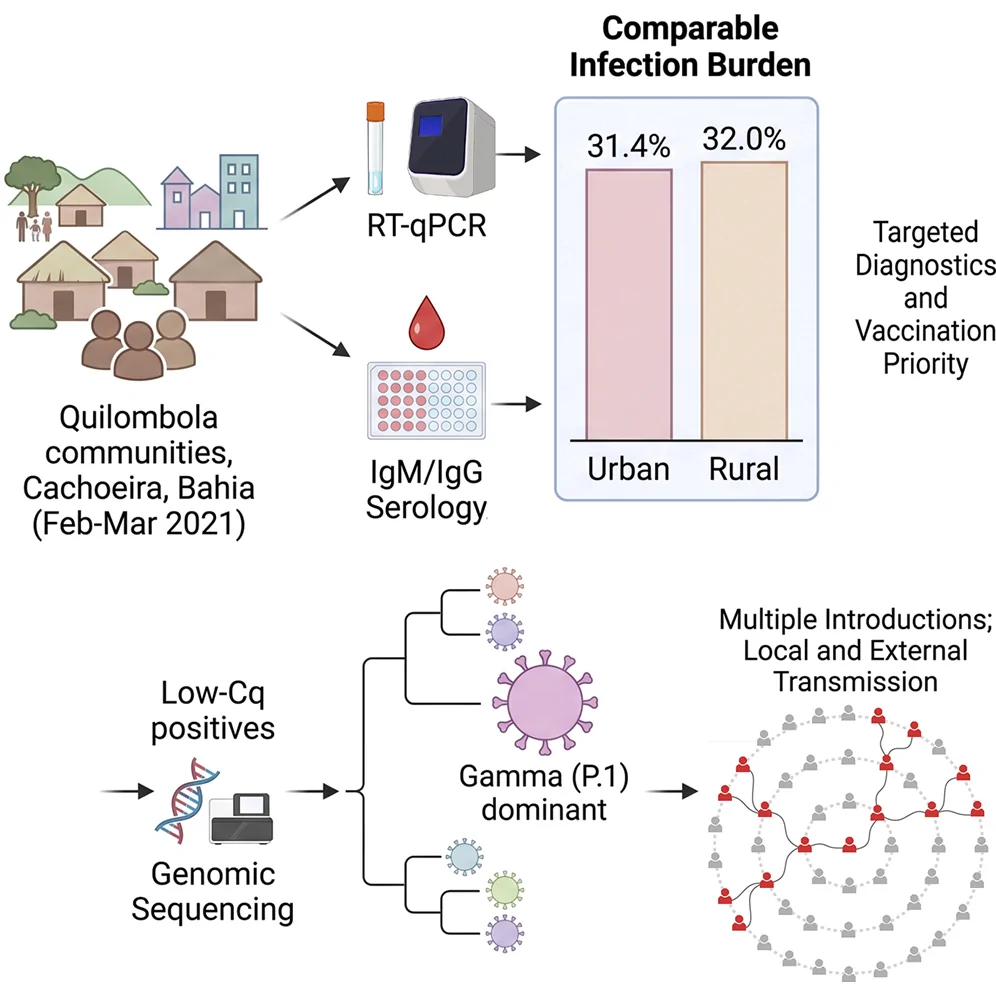

## Teaser

Public health; Omics

## Introduction

SARS-CoV-2, the causative agent of COVID-19, has triggered the most significant pandemic of the 21st century. First identified in December 2019 in Wuhan, Hubei Province, China, the virus rapidly spread worldwide, prompting the World Health Organization (WHO) to declare a public health emergency of international concern on January 30, 2020.^1^ In Brazil, the first major post-introduction shift in SARS-CoV-2 transmission was marked by the emergence and spread of the P.2 lineage in Rio de Janeiro, alongside other minor lineages. However, it was the second wave, driven predominantly by the emergence and nationwide expansion of the P.1 lineage (VOC Gamma), which had a more severe impact on the Brazilian population.^2^

The Brazilian P.1 lineage was identified in Manaus, the capital of Amazonas state, in December of 2020.^3^^,^^4^ The P.1 variant displayed an extensive mutational profile, comprising 21 mutations relative to the ancestral strain, 10 of which affected the spike protein.^2^^,^^5^^,^^6^ The P.1 lineage raised significant concern for health authorities as it is associated with increased transmissibility and disease mortality rates.^7^ From December 2020 to March 2021, during the P.1 dominance, Brazil recorded the highest number of COVID-19-related deaths, according to the WHO (https://covid19.who.int/region/amro/country/br). In this context, the vaccination program, implemented in December 2020, focused initially on groups at higher risk of mortality. It included Indigenous peoples, reflecting recognition of their heightened vulnerability and the need to mitigate disproportionate impacts on Brazil’s first peoples, who have historically faced the enduring consequences of colonization, including forced displacement, social exclusion, and violence.^8^^,^^9^ At the same time, the boundaries of this initial framework also reproduced long-standing patterns of institutional invisibility. Much like Indigenous peoples, Quilombola communities have been historically shaped by colonial and post-colonial processes that produced disproportionate vulnerability and unequal access to care.^10^ Yet they were not included in the initial vaccination plan and were added only later as the eighth priority group.

Quilombola communities are rural settlements established through resistance by Africans subjected to enslavement in Brazil and their descendants.^11^ This historical trajectory has produced persistent structural vulnerability, including social exclusion, inequality, racism, and exposure to violence and criminalization, with direct consequences for access to fundamental rights and public services, particularly healthcare.^10^^,^^12^ The COVID-19 pandemic further intensified these inequities.^13^ In addition to geographic isolation and barriers to reaching urban centers, Quilombola communities faced difficulties in obtaining timely and adequate care, especially as the pandemic strained state health systems.^14^^,^^15^ The municipality of Cachoeira, in Bahia, illustrates this context. Historically, a central hub of the sugar cane economy and the slave trade, it is now home to numerous Quilombola communities. These communities remain vital custodians of cultural heritage and contributors to the local economy, while continuing to experience the effects of long-standing marginalization.^16^

Despite these challenges, the specific impact of SARS-CoV-2 on traditional, unvaccinated, and vulnerable rural populations such as the Quilombolas remains largely unknown. To address this gap, we investigated the dissemination and dynamics of SARS-CoV-2 variants in Quilombola communities in Cachoeira, Bahia. We conducted diagnostic and genomic surveillance during February and March 2021, a period marked by an official state lockdown (Decree N° 20254 from 25/02/2021) and the national peak of Gamma variant prevalence, to evaluate the introduction and potential community transmission of SARS-CoV-2 lineages within these historically neglected populations.

## Results

### SARS-CoV-2 infection patterns in Quilombola communities

Between February and March 2021, 700 participants were recruited from eleven locations in Cachoeira, including urban and rural Quilombola communities (Figure 1A). Oropharyngeal swabs were collected from 693 participants for RT-qPCR testing, and plasma samples were available from 586 participants for IgM and IgG serological analyses (Figure 1B and Table 1). Overall, the study population was predominantly female (61.8%) and middle-aged, with a median age of 43 years (IQR: 32–56 years). No significant differences were observed between urban and rural participants regarding age (median: 44.51 vs. 43.00 years, *p* = 0.582) or sex distribution (63.53% vs. 60.19% female, *p* = 0.491) (Table 1).

**Figure 1:**
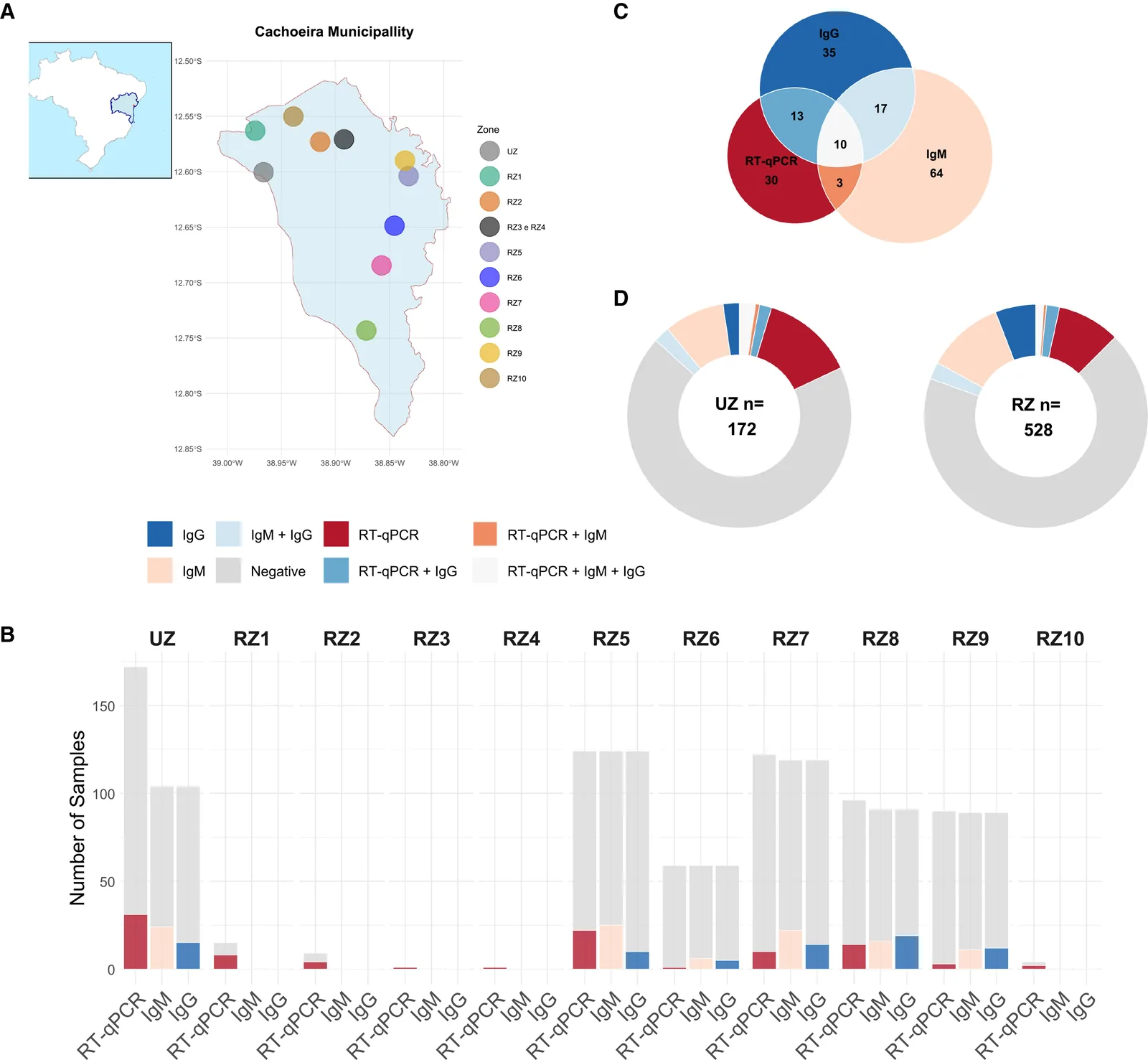
Molecular and serological test results and their intersections among urban and rural zones (A) Geographical map highlighting the sampled zones within the municipality of Cachoeira, Bahia. (B) Diagnostic results for SARS-CoV-2 infection in the study population, by zone. (C) Venn diagram of positive/reactive patients for each test, alone, and combined, among those tested with all three assays. (D) The outer donut chart represents the results of each test, alone or combined.

**Table 1:** Laboratorial and demographical data from Quilombolas individuals

	UZ	RZ	RZ1	RZ2	RZ3 e RZ4^a^	RZ5	RZ6	RZ7	RZ8	RZ9	RZ10
	(*n* = 172)	(*n* = 528)	(*n* = 15)	(*n* = 9)	(*n* = 2)	(*n* = 126)	(*n* = 59)	(*n* = 122)	(*n* = 100)	(*n* = 91)	(*n* = 4)
RT-qPCR detection	18.02%	12.67%	53.33%	44.44%	100%	17.74%	1.69%	8.20%	14.58%	3.33%	50.00%
–	(31/172)	(66/521)	(8/15)	(4/9)	(2/2)	(22/124)	(1/59)	(10/122)	(14/96)	(3/90)	(2/4)
IgM (reagent)	23.08%	16.60%	–	–	–	20.16%	10.17%	18.49%	17.58%	12.36%	–
–	(24/104)	(80/482)	–	–	–	(25/124)	(6/59)	(22/119)	(16/91)	(11/89)	–
IgG (reagent)	14.42%	12.45%	–	–	–	8.06%	8.47%	11.76%	20.88%	13.48%	–
–	(15/104)	(60/482)	–	–	–	(10/124)	(5/59)	(14/119)	(19/91)	(12/89)	–
IgG, IgM and/or RT-qPCR	31.4%	32.0%	53.3%	44.4%	100.0%	38.8%	16.9%	30.1%	35.3%	20.4%	50.0%
–	(54/172)	(169/528)	(8/15)	(4/9)	(2/2)	(50/129)	(10/59)	(37/123)	(36/102)	(20/98)	(2/4)
Age median (years)	44.51	43	23	23	33	50	41	41	42	44	39
IQR	29–57.5	32–55	18–26	18–41	33	36–66	34.5–60.5	29–47	34–50	35–57	35–44.5
Sex feminine	63.53%	60.19%	60%	55.56%	0	61.29%	49.15%	62.30%	71.72%	61.54%	50%
–	(108/170)	(325/540)	(9/15)	(5/9)	(0/1)	(76/124)	(29/59)	(76/122)	(71/99)	(56/91)	(2/4)

a RZ3 and RZ4 were grouped due to geographical proximity and low number of samples.

Among participants with evidence of SARS-CoV-2 infection or exposure detected by at least one assay (*n* = 172), IgM was the most frequently identified marker (94/172, 54.7%), followed by IgG (75/172, 43.6%) and RT-qPCR positivity (56/172, 32.6%). Despite the relatively high reactivity/positivity rates observed across individual assays, concordance between methods was limited, with only 10 of 172 participants (5.8%) testing positivity by RT-qPCR, IgM, and IgG simultaneously (Figure 1C). This pattern is consistent with the distinct biological windows captured by molecular and serological assays and suggests heterogeneity in the timing of infection and immune responses across the surveyed people seeking care in Quilombola communities.

These patterns were then evaluated across locations and after aggregation into the two primary settings (urban vs. rural). Demographic assessment suggested variability in age and sex distributions across the urban and rural Quilombolas zones. A similar pattern was observed for SARS-CoV-2 detection using combined RT-qPCR and serology, with variability across the studied zones. However, when stratified into two major geographic groups, urban (UZ, *n* = 172) and aggregated rural Quilombola zones (RZ, *n* = 528), age and sex distributions as well as overall test positivity by RT-qPCR and/or serology were comparable between settings (*p* > 0.05) (Figure 1D and Table 1).

Although overall test reactivity/positivity was comparable between urban and rural settings, the infection burden detected among individuals participating in the testing campaign was substantial and contrasted with municipal surveillance reports. Using a composite definition of infection based on RT-qPCR and/or serological test positivity/reactivity, 32% of Quilombola participants tested during the outbreak investigation were classified as exposed within one year of the first confirmed SARS-CoV-2 cases in Brazil, suggesting viral circulation among the population reached by this public health intervention (Figure 1D and Table 1). While the non-probabilistic, care-seeking nature of the sample limits the generalizability of these findings and precludes estimation of community-level prevalence, the high proportion of positive individuals nonetheless indicates a considerable burden of SARS-CoV-2 infection among those evaluated during the campaign and highlights the vulnerability of these historically underserved communities during the pandemic.

In contrast, municipal surveillance data from Cachoeira, a municipality with an estimated population of 33,567 inhabitants in 2020–2021, reported only 808 notified COVID-19 cases and nine deaths during the same period (Figure 2A). Although direct comparisons should be interpreted with caution given the facility-based sampling strategy employed in this study, the marked discrepancy between official notifications and the frequency of infections detected in our study may reflect underdiagnosis, barriers to healthcare access, and limited availability of timely testing in Quilombola communities. Such factors may have contributed to an underestimation of the true impact of SARS-CoV-2 in these vulnerable populations.

**Figure 2:**
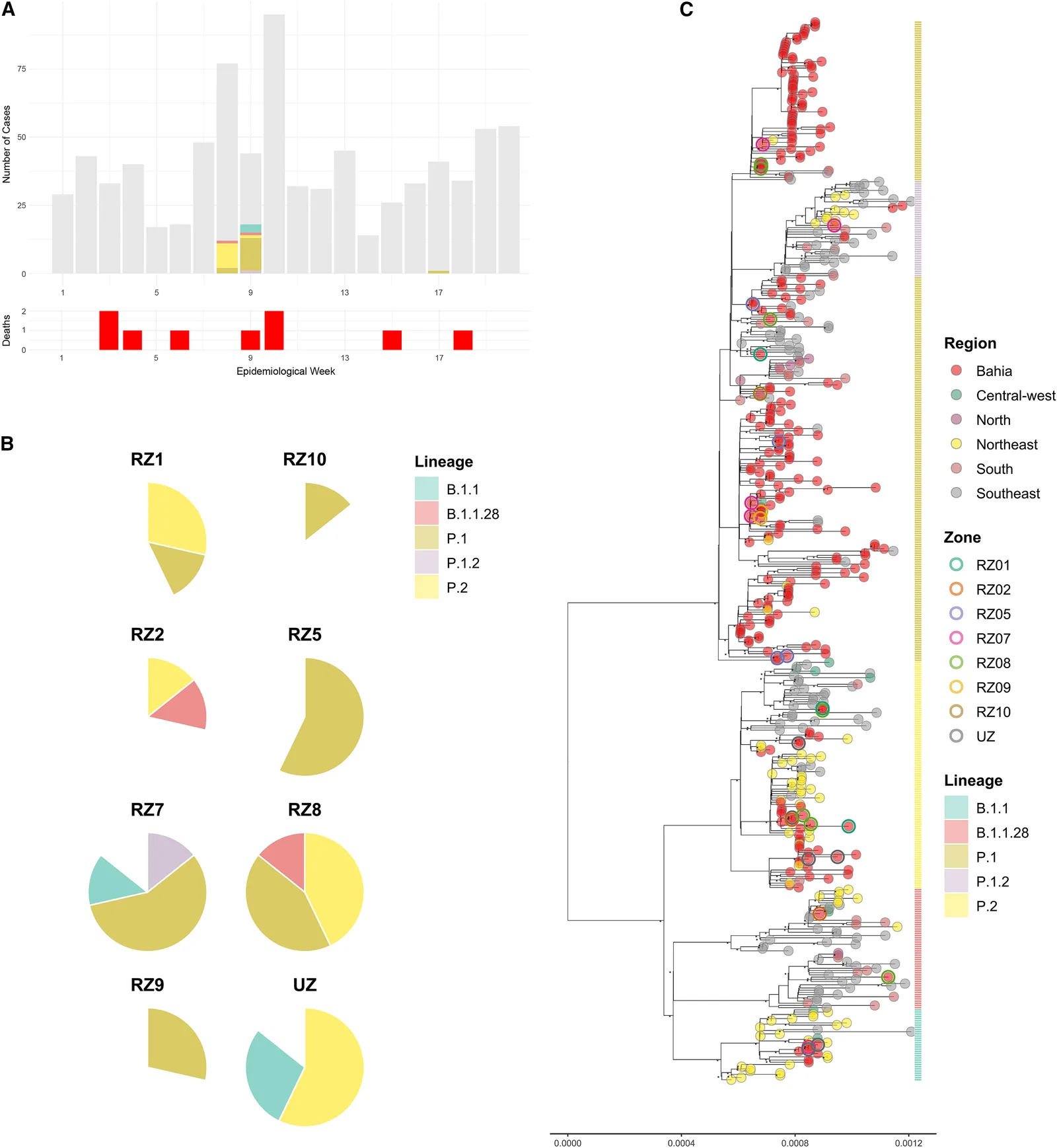
Genomic surveillance of SARS-CoV-2 in Cachoeira, Brazil (A) Epidemiological summary for the municipality, displaying the number of confirmed cases, deaths, and the breakdown of lineages identified throughout the study period. (B) Stacked chart showing the proportion of circulating SARS-CoV-2 lineages over time within each studied zone. (C) Phylogenetic reconstruction based on the maximum likelihood method, depicting the evolutionary relationships of the SARS-CoV-2 lineages sequenced in this study.

### Genomic characterization of SARS-CoV-2 sequences

To characterize the genomic diversity of SARS-CoV-2 circulating in the studied Quilombola communities and to infer patterns of viral introduction and spread between settings, we performed whole-genome sequencing and phylogenetic analyses. A total of 39 viral sequences were generated in this study, including 7 from urban areas and 32 from 7 rural regions. Among these, three samples were classified as lineage B.1.1, two as B.1.1.28, 23 as lineage P.1, and 11 as lineage P.2. The relative frequency of these lineages by location is shown in Figure 2B. For the phylogenetic analysis, 8 of the 39 sequences were excluded because they contained more than 5% ambiguous nucleotides (Ns). The B.1.1 dataset comprised 812 genomic sequences, while the B.1.1.28 dataset included 4,575 sequences. Because P.1.2 is a sublineage of Gamma, P.1 and P.1.2 reference sequences were analyzed jointly. The resulting dataset contained 43,074 sequences and was randomly subsampled to 7,000 sequences. The P.2 lineage comprised 4,521 sequences. These datasets provide a comprehensive overview of the genomic diversity and distribution of key SARS-CoV-2 variants analyzed in this study (https://doi.org/10.6084/m9.figshare.33094550). To assess the broader context of viral circulation in the region, a final dataset of 485 sequences was analyzed (Figure 2C).

Phylogenetic reconstruction revealed multiple independent introduction events, observed by the presence of sequences from Quilombola communities is several branches, primarily originating from Bahia, with additional dissemination from southeast Brazil. Two well-supported monophyletic clusters (bootstrap >80%), assigned to the P.1 and P.2 lineages, consisted exclusively of sequences from the same Quilombola communities, suggesting autochthonous transmission. Two additional clusters, belonging to the B.1.1 and P.2 lineages, included sequences from both rural and urban settings (bootstrap >80%). The remaining three clusters, associated with P.1 and P.2, comprised only rural samples from distinct Quilombola communities. Collectively, these five clusters suggest epidemiological connectivity and viral exchange between rural and urban Quilombola zones.

Phylogenetic reconstruction was consistent with multiple independent SARS-CoV-2 introduction events. Putative introductions were inferred when study sequences clustered within distinct, well-supported clades containing genomes sampled outside the Quilombola communities. These findings are consistent with independent viral introductions but should not be interpreted as direct evidence of transmission direction. Most inferred introductions were linked to sequences previously reported from Bahia and, to a lesser extent, from Southeast Brazil. Two well-supported monophyletic clusters (bootstrap >80%), assigned to the P.1 and P.2 lineages, consisted exclusively of sequences from the same Quilombola communities, suggesting local transmission. Two additional clusters, belonging to the B.1.1 and P.2 lineages, included sequences from both rural and urban settings (bootstrap >80%), indicating potential epidemiological connectivity between these populations. The remaining three clusters, associated with P.1 and P.2, comprised only rural samples from distinct Quilombola communities. However, these findings should be interpreted with caution, as genome recovery was limited to a subset of RT-qPCR-positive samples with sufficient viral load, and the sequenced genomes may not fully represent the diversity of lineages circulating in the study area. Therefore, the absence of a given lineage in a specific rural or urban area should not be interpreted as evidence of its true absence, but rather as a consequence of limited sampling and sequencing coverage, together with the low number of sequences available from urban areas. These clusters were defined based on phylogenetic relatedness and bootstrap support (>80%) and should be interpreted as groups consistent with potential epidemiological linkage rather than confirmed transmission clusters.

## Discussion

This study documents a complex and uneven spread of SARS-CoV-2 among Quilombola communities in Bahia, Brazil, that occurred soon after transmission became established in major urban centers. It provides the first characterization of lineage distributions in these communities. Within a year of the virus’s arrival, at least 32% of individuals seeking care from these populations showed evidence of SARS-CoV-2 infection or exposure, based on the combined detection of RT-qPCR, IgM, and IgG markers. This approach was adopted to provide a broader estimate of the overall burden of infection, capturing active or recent infections (RT-qPCR positivity and IgM reactivity) as well as previous exposure (IgG reactivity). However, these markers reflect distinct biological stages of infection and differ in their windows of detection and diagnostic performance, which should be considered when interpreting the findings. In addition, false-positive and false-negative results may have influenced estimates of infection burden, and the timing of sample collection relative to infection may have affected the detection of molecular and serological markers.

Importantly, vaccination was not a confounding factor in the interpretation of serological results, as samples were collected between February and March 2021, before COVID-19 vaccines became available in these Quilombola communities. Therefore, IgM and IgG reactivity are likely to reflect natural SARS-CoV-2 infection rather than vaccine-induced immune responses. Nevertheless, the high frequency of positive tests suggests substantial and rapid exposure to SARS-CoV-2 in these communities, indicating that vulnerable populations such as Quilombola groups may benefit from early inclusion in public health measures, including vaccination programs.^17^ The wide variation in RT-qPCR positivity rates indicates non-uniform viral circulation, likely due to localized outbreaks. While our data on urban versus rural zones align with national trends of unequal spread, contrasting reports exist, potentially due to methodological differences such as the use of immunochromatography in community-based surveys versus RT-qPCR in care-seeking populations.^18^^,^^19^ These discrepancies reinforce the necessity of combining molecular and serological data to fully capture transmission dynamics, as serology reveals a cumulative incidence, and of considering demographic factors that influence both susceptibility and healthcare-seeking behavior.^20^^,^^21^

Our genomic analysis suggests a transmission landscape shaped by multiple SARS-CoV-2 introductions, primarily from within Bahia and Brazil’s Southeast region, followed by local dissemination, consistent with previous reports from Bahia State.^22^ Phylogenetic reconstruction identified well-supported monophyletic clusters of P.1 (Gamma) and P.2 within individual communities, a pattern compatible with local transmission and previously reported in other vulnerable populations.^23^ In addition, clusters containing both rural and urban sequences suggest possible epidemiological links between these settings. The predominance of the highly transmissible P.1 variant among the recovered genomes is noteworthy given its documented association with increased transmissibility and immune evasion.^3^

These observations should be interpreted considering important limitations. Genome sequencing was restricted to RT-qPCR-positive samples with sufficiently low Cq values, favoring samples with higher viral loads and potentially introducing selection bias. Furthermore, only a subset of positive samples yielded genomes suitable for analysis, limiting the representativeness of the genomic dataset. Consequently, the recovered genomes may not capture the full spectrum of SARS-CoV-2 lineages circulating in these communities, and the absence of a given lineage in a specific location should not be interpreted as evidence of its true absence. Furthermore, phylogenetic inferences were based on available genomes and should be interpreted as evidence consistent with viral introductions and local transmission patterns rather than definitive proof of transmission direction or epidemiological linkage. These limitations are particularly relevant given the historically low level of genomic surveillance in the region.^24^^,^^25^ Limited sampling may also explain the absence of some lineages in certain settings despite their documented circulation elsewhere in Bahia.

In addition, this study has several limitations that should be considered when interpreting the findings. First, its cross-sectional design provides only a snapshot of SARS-CoV-2 circulation during the study period and does not allow inference of temporal or causal relationships. Second, participants were recruited through a public health testing campaign implemented in response to a COVID-19 outbreak and were identified by local health services based on suspected infection, resulting in a non-probabilistic convenience sample. Therefore, the observed test positivity should be interpreted as the infection burden among the Quilombola population rather than as an estimate of community-level prevalence. Although all eligible individuals referred during the campaign were included, the sample may not be representative of the entire Quilombola population of the municipality. Additionally, detailed individual-level epidemiological information, such as travel history, household characteristics, occupational exposures, and contact networks, was not systematically collected, limiting the investigation of specific transmission pathways. Finally, the composite infection outcome, based on RT-qPCR and/or serological test positivity, was intended to capture both current and previous SARS-CoV-2 infections; however, the distinct biological meanings and temporal windows represented by these markers should be considered when interpreting the results.

The high early test positivity and complex transmission dynamics, featuring both isolated community-specific spread and inter-zone connectivity, suggests the presence of both localized and interconnected transmission patterns in Quilombola populations. Evidence of rapid variant spread and persistent local transmission clusters, occurring in a context of insufficient genomic surveillance and inadequate vaccination coverage, highlights a structural failure that perpetuates historical health inequities, as has been documented in Brazil.^10^^,^^26^^,^^27^ This study aligns with the guidelines of the Brazilian Ministry of Health, contributing to etiological confirmation and to the generation of evidence necessary to support technical decision-making and public health policy formulation.^8^ In the context of SARS-CoV-2 and other respiratory viruses, as well as emerging arboviruses, laboratory confirmation and, when appropriate, molecular characterization (including lineage assignment and identification of introduction events) provide critical support for decisions related to active case finding, testing strategies, the organization of sentinel surveillance, rational resource allocation, risk communication, and the implementation of measures within health services.

Furthermore, the data generated by this study provide a robust technical foundation for defining goals, indicators, and operational workflows for regional surveillance, with an emphasis on prioritizing vulnerable territories, such as the Recôncavo region of Bahia, as well as other areas of Brazil characterized by access barriers, limited connectivity, and complex logistical challenges in health service delivery. In addition, many Quilombola communities, including those represented here, maintain close interactions with surrounding ecosystems through agriculture, fishing, extractive activities, and forest-edge livelihoods, which may increase exposure to a broader range of zoonotic and environmentally associated pathogens and heighten the potential to detect previously unrecognized spillover events. Taken together, these findings highlight the imperative for equitable and sustained surveillance, coupled with inclusive public health policies, to ensure accurate mapping of transmission pathways and adequate protection of marginalized populations, particularly in socially vulnerable and hard-to-reach territories.

This study provides important insights into the complex dissemination of SARS-CoV-2 in Quilombola communities in Cachoeira, Bahia. Our analysis revealed uneven viral spread between urban and rural areas, with a notable predominance of the P.1 (Gamma) variant. The diversity of lineages identified in the recovered genomes highlights the value of genomic surveillance for understanding SARS-CoV-2 circulation in underrepresented populations. However, given the limited number of genomes obtained and the potential sampling bias associated with sequencing success, these findings should be interpreted as a partial representation of the viral diversity circulating in the study area. Public policies must prioritize these historically marginalized populations by ensuring access to diagnosis, treatment, and vaccination. Inclusive and targeted actions are essential to mitigate the impacts of future health emergencies in these communities.

### Limitations of the study

Sequencing only RT-qPCR-positive samples with low Cq introduced selection bias, and incomplete genome recovery limits representativeness, so circulating lineages may be underrepresented and absence of a lineage should not be taken as true absence. Phylogenetic findings suggest introductions and local transmission but cannot prove transmission direction or epidemiological linkage, especially given historically low genomic surveillance and limited sampling. The cross-sectional design offers only a snapshot and precludes temporal or causal inference. Participants formed a non-probabilistic convenience sample from an outbreak testing campaign based on suspected infection, so positivity reflects infection burden among those tested rather than community prevalence, and the sample may not represent the entire Quilombola population. Detailed individual-level epidemiological data were not systematically collected, limiting investigation of transmission pathways. Finally, the composite RT-qPCR/serology outcome captures current and past infections, but these markers have distinct biological meanings and temporal windows that must be considered.

## Resource availability

### Lead contact

Requests for further information and resources should be directed to and will be fulfilled by the lead contact, Filipe Ferreira de Almeida Rego (filipe.rego@fiocruz.br).

### Materials availability

This study did not generate any new unique reagents or other materials that require availability or distribution.

### Data and code availability

• All consensus SARS-CoV-2 genome sequences generated in this study have been deposited in GISAID under accession codes (EPI_ISL_2663261, EPI_ISL_11780216, EPI_ISL_11780214, EPI_ISL_2663264, EPI_ISL_2663265, EPI_ISL_10580297, EPI_ISL_11780215, EPI_ISL_2663267, EPI_ISL_2663268, EPI_ISL_2663269, EPI_ISL_10580299, EPI_ISL_10101582, EPI_ISL_2663271, EPI_ISL_10580301, EPI_ISL_10101583, EPI_ISL_2663273, EPI_ISL_2663274, EPI_ISL_2663276, EPI_ISL_2663277, EPI_ISL_2663257, EPI_ISL_2663279, EPI_ISL_2663280, EPI_ISL_2663281, EPI_ISL_10101584, EPI_ISL_10580302, EPI_ISL_10580298, EPI_ISL_2663282, EPI_ISL_2663283, EPI_ISL_2663284, EPI_ISL_2663285, EPI_ISL_2663286, EPI_ISL_2663287, EPI_ISL_2663288, EPI_ISL_2663289, EPI_ISL_2663290, EPI_ISL_10580296, EPI_ISL_2663293, EPI_ISL_10580300, EPI_ISL_6908829). Curated sequence datasets, phylogenetic tree files, tabulated epidemiological data, and lineage assignment results are publicly available at Figshare: https://doi.org/10.6084/m9.figshare.33094550.
• All original analysis scripts and code used in this study are publicly available at Figshare: https://doi.org/10.6084/m9.figshare.33094550.
• Any additional information required to reanalyze the data reported in this study is available from the lead contact upon request.

## Acknowledgments

This work was funded by the Fiocruz COVID-19 Genomic Surveillance Network, the 10.13039/501100004735Natural Science Foundation of Hunan Province (2024JJ2069) and the 10.13039/501100001809National Nature Science Foundation of China (92569203). The authors wish to thank the GISAID team and all the submitters to the EpiCoV database. The authors acknowledge the availability of SARS-CoV-2 sequences in the GISAID database and all contributors who have improved genomic surveillance in Brazil https://doi.org/10.55876/gis8.260109cs). L.P.M. was supported by 10.13039/501100002322Coordination for the Improvement of Higher Education Personnel (10.13039/501100002322CAPES; financial code 001).

## Author contributions

The study was conceived and designed by R.K., H.P.d.S.F., J.S.N., F.V., and J.B.L.R. F.F.d.A.R. and L.A.S. performed the statistical analysis, interpreted the results, and wrote the first draft. R.K., C.I.d.O., and H.P.d.S.F. managed the study and coordinated data collection. J.S.N., F.V., J.B.L.R., M.S.C., and L.M. were responsible for laboratory sample analysis, sample management, and interpretation of laboratory results. P.T.V.F., V.d.A.O., C.F., and J.B.J. were responsible for collecting and curating regional epidemiological data. G.L., R.K., and H.P.d.S.F. reviewed and edited the manuscript. All authors contributed to the final revision of the manuscript and approved the final draft.

## Declaration of interests

The authors declare no competing interests.

## Declaration of generative AI and AI-assisted technologies in the manuscript preparation process

During the preparation of this work the authors used GPT-5.6 Luna in order to assist with English language editing, including grammar, spelling, clarity, and readability. After using this tool/service, the authors reviewed and edited the content as needed and take full responsibility for the content of the publication.

## STAR★Methods

### Key resources table

**Table:** 

REAGENT or RESOURCE	SOURCE	IDENTIFIER
**Biological samples**		

Oropharyngeal swab samples from Quilombola individuals	This paper	N/A
Plasma samples from Quilombola individuals	This paper	N/A

**Chemicals, peptides, and recombinant proteins**		

5 Hot Start High-Fidelity DNA Polymerase	New England BioLabs	Cat# M0493L

**Critical commercial assays**		

Allplex™ SARS-CoV-2 Assay	Seegene	Cat# RV10248X
TR DPP® COVID-19 Ag – Bio-Manguinhos	Bio-Manguinhos/Chembio Diagnostics Systems, Inc.	MS Registration: 80142170047
SuperScript IV First-Strand Synthesis System	Thermo Fisher Scientific	Cat# 18091050
Illumina DNA Prep kit	Illumina	Cat# 20060059 (96 samples)/20060060 (24 samples)

**Deposited data**		

Consensus SARS-CoV-2 genome sequences: EPI_ISL_2663261, EPI_ISL_11780216, EPI_ISL_11780214, EPI_ISL_2663264, EPI_ISL_2663265, EPI_ISL_10580297, EPI_ISL_11780215, EPI_ISL_2663267, EPI_ISL_2663268, EPI_ISL_2663269, EPI_ISL_10580299, EPI_ISL_10101582, EPI_ISL_2663271, EPI_ISL_10580301, EPI_ISL_10101583, EPI_ISL_2663273, EPI_ISL_2663274, EPI_ISL_2663276, EPI_ISL_2663277, EPI_ISL_2663257, EPI_ISL_2663279, EPI_ISL_2663280, EPI_ISL_2663281, EPI_ISL_10101584, EPI_ISL_10580302, EPI_ISL_10580298, EPI_ISL_2663282, EPI_ISL_2663283, EPI_ISL_2663284, EPI_ISL_2663285, EPI_ISL_2663286, EPI_ISL_2663287, EPI_ISL_2663288, EPI_ISL_2663289, EPI_ISL_2663290, EPI_ISL_10580296, EPI_ISL_2663293, EPI_ISL_10580300, EPI_ISL_6908829	This paper	https://doi.org/10.6084/m9.figshare.33094550

**Oligonucleotides**		

18 primer pairs for SARS-CoV-2 genome amplification	Resende et al.^28^	N/A

**Software and algorithms**		

ViralFlow v.0.1.0	Dezordi et al.^29^	https://github.com/dezordi/ViralFlow
Pangolin v.4.3.1	O’Toole et al.^30^	https://github.com/cov-lineages/pangolin;
Nextclade v.3.15.3	Nextstrain	https://clades.nextstrain.org
Geneious Prime 2025.2.2	Biomatters	https://www.geneious.com/
ViralMSA v.1.1.45	Moshiri^31^	https://github.com/niemasd/ViralMSA
AliView v.1.26	Larsson^32^	https://ormbunkar.se/aliview/
IQ-TREE v.1.6.12	Minh et al.^33^	http://www.iqtree.org
UFBoot2	Hoang et al.^34^	http://www.iqtree.org
ModelFinder (in IQ-TREE 1.5.4)	Kalyaanamoorthy et al.^35^	http://www.iqtree.org
ggtree v.3.16.3	Xu et al.^36^	https://bioconductor.org/packages/ggtree/; RRID:SCR_ [inserir]
readxl v.1.45.5	CRAN	https://cran.r-project.org/package=readxl
dplyr v.1.2.0	CRAN	https://cran.r-project.org/package=dplyr
lubridate v.1.9.5	CRAN	https://cran.r-project.org/package=lubridate
ggplot2 v.4.0.2	CRAN	https://cran.r-project.org/package=ggplot2
tidyr v.1.3.2	CRAN	https://cran.r-project.org/package=tidyr
RColorBrewer v.1.1–3	CRAN	https://cran.r-project.org/package=RColorBrewer
patchwork v.1.3.2	CRAN	https://cran.r-project.org/package=patchwork
R scripts for data visualization	This paper	https://doi.org/10.6084/m9.figshare.33094550

### Experimental model and study participant details

#### Participant selection

This cross-sectional study was conducted in the municipality of Cachoeira, Bahia, Brazil, during the second wave of SARS-CoV-2 infections, between February and March 2021. The study was based on a municipal COVID-19 testing campaign implemented in response to a SARS-CoV-2 outbreak affecting Quilombola communities. The initiative originated from a request for assistance from the affected communities and local health authorities. In collaboration with Quilombola leaders, the Municipal Health Secretariat, the Municipal Epidemiological Surveillance Center (CIEVS), community health agents, and the research team, a testing strategy was developed and implemented to diagnose SARS-CoV-2 infections and support healthcare actions within Brazil’s Unified Health System (SUS).

Participants consisted of suspected COVID-19 cases identified by local health services and referred for molecular and serological testing through the Molecular Surveillance Platform (PVM), resulting in a non-probabilistic convenience sample. Inclusion criteria comprised Quilombola individuals presenting with influenza-like illness (ILI), defined as an acute respiratory condition with at least two of the following symptoms: fever (reported or measured), chills, sore throat, headache, cough, runny nose, olfactory dysfunction (anosmia), or gustatory dysfunction (ageusia). All eligible Quilombola individuals tested for SARS-CoV-2 during the study period were included in the analysis. No exclusion criteria were applied.

The study included 700 participants, of whom 528 resided in rural areas and 172 in urban areas. Age was available for 697 participants, with a median of 43 years (interquartile range, 31–56 years; range, 2–86 years). Recorded sex was female for 433 participants (61.9%) and male for 264 participants (37.7%); sex information was unavailable for three participants (0.4%). Age information was also unavailable for three participants. The study population comprised members of Quilombola communities; individual genetic ancestry was not assessed.

As this was an observational study, participants were not randomly allocated to experimental groups, and no research interventions were administered. Analytical groups were defined according to participants’ area of residence (rural or urban) and molecular and serological test results. Associations of sex or gender with the study outcomes were not evaluated. Therefore, the findings do not establish whether molecular detection, serological reactivity, or viral genomic patterns differed according to sex or gender, which represents a limitation of the study.

#### Sample collection and public health response

Sample collection was conducted jointly by local health authorities, community health agents, and members of the research team, while molecular and serological analyses were performed by the research team using a diagnostic platform established to support SUS activities. Test results were subsequently made available through the national Laboratory Environment Manager (Gerenciador de Ambiente Laboratorial, GAL) system and communicated to participants by community health agents, who also facilitated access to healthcare and follow-up when needed.

#### Ethics approval and informed consent

This study was conducted in accordance with the ethical principles of the Declaration of Helsinki and Brazilian regulations governing research involving human participants. Priority was given to the public health response and to ensuring appropriate care for affected individuals. Only after these measures were established were community representatives and local institutions consulted regarding the research use of the anonymized data generated during the testing campaign. Community leaders formally endorsed the initiative through letters of support and agreed to the secondary use of these data for research purposes, supporting respectful and equitable engagement with the participating Quilombola communities.

The research protocol, including the secondary use of anonymized surveillance data generated during the community-based public health response, was reviewed and approved by the Research Ethics Committee of the Instituto Gonçalo Moniz (CAAE: 78550824.8.0000.0040). No additional biological samples were collected, and no questionnaires or other research procedures were administered to participants. Because the study involved only secondary analysis of anonymized data, the Ethics Committee waived the requirement for individual written informed consent.

### Method details

#### Study design

##### SARS-CoV-2 detection

Swab samples were processed, and COVID-19 diagnosis was performed by RT-qPCR using the Kit Allplex SARS-CoV-2 Assay (Seegene) at the Molecular Surveillance Platform at Gonçalo Moniz Institute (IGM). In addition, serological screening was performed using plasma samples using the TR DPP® COVID-19 Ag - Bio-Manguinhos, which analyzed IgM and IgG.

##### Sequencing and analysis

To sequence the positive samples, viral RNA was extracted from all samples with an RT-qPCR quantification cycle <25 (Cq). An *in-house* protocol from LVRS-FIOCRUZ-RJ was used to perform cDNA using SuperScript IV First-Strand Synthesis System (Thermo Fisher Scientific) and PCR using Q5 Hot Start High-Fidelity DNA Polymerase (New England BioLabs) with 18 described primer pairs.^28^ Library preparation was performed using the DNA Prep kit (Illumina), and sequencing was performed on the Illumina MiSeq platform. The obtained sequences were submitted to the ViralFlow v.0.1.0, with default pipeline available at GitHub (https://github.com/dezordi/ViralFlow).^29^

##### Lineage characterization and phylogenetic reconstruction

All consensus genome sequences were classified using the Phylogenetic Assignment of Named Global Outbreak Lineages (Pangolin) tool v.4.3.1.^30^ The mutation profile was accessed via the Nextclade platform 3.15.3 (https://clades.nextstrain.org). To estimate evolutionary relationships, we first built a dataset comprising sequences retrieved from the Global Initiative on Sharing Avian Influenza Data (https://www.gisaid.org/), including all Brazilian sequences of the assigned pangolin lineages with complete data and complete genomes; sequences with low coverage were excluded. Datasets larger than 7000 sequences were subsampled to ensure reproducibility. Sequences were randomly subsampled in Geneious 2025.2.2 using simple random sampling without replacement. Sequence indices were selected uniformly at random until the target sample size was reached. The alignment was performed with ViralMSA v.1.1.45,^31^ and the manual edition was performed with AliView v.1.26.^32^

Phylogenetic analyses were initially performed for each lineage, after which clades containing the study samples were extracted to reconstruct a phylogenetic tree encompassing all lineages. Maximum-Likelihood phylogenetic trees were built through IQ-TREE v.1.6.12,^33^ using the ultrafast bootstrap approximation with 1000 pseudoreplicates.^34^ The base-substitution model was selected using ModelFinder implemented in IQ-TREE 1.5.4.^35^ The phylogenetic signal was evaluated by likelihood mapping implemented in IQ-TREE. The trees were viewed and edited using the ggtree v.3.16.3 package from R in addition to readxl v.1.45.5, dplyr v.1.2.0, lubridate v.1.9.5, ggplot2 v.4.0.2, tidyr v.1.3.2, RColorBrewer v.1.1–3 and patchwork v.1.3.2.^36^ The final tree was reconstructed using all sequences from this study, along with at least 10 of the closest sequences from the previous analysis with bootstrap support, following the pipeline above.

### Quantification and statistical analysis

Demographic information, including age and sex, was collected at the time of sample collection. Exposure history, occupation, household size, and mobility data were evaluated collected during the study. COVID-19 vaccination status was not evaluated because vaccines were not yet available to this population during the study period. Blood samples and oropharyngeal swabs were collected from participants residing in the urban area of Cachoeira (urban zone, UZ) and in ten Quilombola communities, hereafter referred to as rural zones (RZ): Capoeiruçu (RZ1), Alecrim (RZ2), Povoado do Saco (RZ3), Murutuba (RZ4), Opalma (RZ5), Kaonge (RZ6), Santiago do Iguape (RZ7), São Francisco do Paraguaçu (RZ8), Tabuleiro da Vitória (RZ9), and Belém (RZ10). The study database was carefully reviewed for consistency, and no duplicate participants were identified. Age was analyzed using the Mann-Whitney U test, whereas categorical variables (sex, RT-qPCR positivity, IgM, and IgG reactivity) were compared using Pearson’s χ^2^ test or Fisher’s exact test when expected cell counts were <5. Statistical significance was defined as a two-sided *p* value < 0.05.
